# Xpert MTB/RIF Ultra performance on bronchial specimens in diagnosing pulmonary tuberculosis in Vancouver, Canada

**DOI:** 10.1099/jmm.0.002022

**Published:** 2025-06-06

**Authors:** Cole Schonhofer, Jennifer Bilawka, Scott Apperley, Christopher F. Lowe, Michael Payne, Nancy Matic, Victor Leung, Marc G. Romney, Patrick Tang, Aleksandra Stefanovic

**Affiliations:** 1 Department of Pathology and Laboratory Medicine, University of British Columbia, Vancouver, Canada; 2 Division of Medical Microbiology and Virology, St. Paul’s Hospital, Providence Health Care, Vancouver, Canada; 3 Division of Respiratory Medicine, St. Paul’s Hospital, Providence Health Care, Vancouver, Canada

**Keywords:** *Mycobacterium tuberculosis*, GeneXpert MTB/RIF Ultra, bronchoscopy

## Abstract

**Introduction.** In cases where sputum is non-diagnostic or unavailable, bronchoscopy can yield high-value respiratory samples for tuberculosis (TB) diagnosis. Whilst *Mycobacterium tuberculosis* (MTB) *complex* culture remains the gold standard, molecular assays such as Xpert MTB/RIF Ultra (Xpert Ultra) are increasingly being used for rapid diagnosis.

**Gap Statement.** Xpert Ultra is increasingly used for TB diagnosis and has been extensively evaluated on sputum specimens, but assessment of performance on bronchoscopy samples is more limited.

**Aim.** To retrospectively evaluate the performance of Xpert Ultra on bronchoscopy specimens in comparison to culture in a low-incidence, high-resource setting.

**Methodology.** Patients with a clinical suspicion of TB, who had non-diagnostic sputum or limited sputum production and underwent bronchoscopy between March 2019 and October 2023, were included in the study. Bronchoscopy specimens comprised bronchoalveolar lavages, bronchial washings and endobronchial ultrasound lymph node tissue biopsies. All included specimens underwent acid-fast bacilli (AFB) smear, mycobacterial culture and Xpert Ultra. Positive MTB culture was considered the reference standard for TB diagnosis.

**Results.** One hundred thirty-five bronchoscopy samples from 126 patients were included. Cultures were positive for MTB in 47 out of 126 (37.3%) of included patients. Overall, positive percent agreement (PPA) and negative percent agreement (NPA) of Xpert Ultra to MTB culture were 93.6% and 98.7%, respectively. In 19 AFB smear-positive cases, Xpert Ultra had 100% PPA and NPA, whilst in 28 smear-negative cases, PPA and NPA were 89.3% and 98.6%, respectively. On average, positive culture results were available after 15.2 days of incubation (range, 5–42 days) versus 24 h for Xpert Ultra. Xpert Ultra PCR cycle threshold values correlated strongly with AFB-smear grade and time-to-culture positivity.

**Conclusion.** Xpert Ultra performed on specimens collected via bronchoscopy demonstrated excellent agreement with culture, even in smear-negative cases. Our results support the use of the Ultra on bronchoscopy specimens for accurate and rapid TB diagnosis in a low-incidence setting.

## Introduction

Globally, tuberculosis (TB) is estimated to infect 25% of the population, with 10.6 million active infections and 1.3 million deaths in 2022 [1]. In Canada, the incidence of active TB was low at 4.8 cases per 100,000 population in 2021, with most cases occurring in foreign-born individuals and pulmonary TB being the most common presentation [2]. Traditionally, laboratory diagnosis has relied on the growth of *Mycobacterium tuberculosis* (MTB) *complex* in culture for identification and antibiotic susceptibility testing. Although culture represents the gold standard for diagnosis, it typically takes 2–8 weeks for MTB to grow [3]. Benefits of earlier diagnosis include expedited initiation of therapy with decreased morbidity, mortality and transmission [4]. Direct examination of smears for acid-fast bacilli (AFB) is fast and inexpensive but is insensitive and unable to distinguish between MTB and non-tuberculous mycobacteria (NTM). In particular, smear-negative TB infections have historically been challenging to diagnose in a timely fashion.

Over the past decade, PCR-based methods have been widely adopted and recommended for accurate and rapid diagnosis of TB [5]. One such method endorsed by the World Health Organization is GeneXpert MTB/RIF (rifampin) Ultra (Xpert Ultra) (Cepheid, USA). This molecular assay targets the *IS6110* and *IS1081* gene regions for MTB detection as well as mutations within the *rpoB* gene to screen for RIF resistance [6,7]. A meta-analysis including different populations and specimen types showed improved performance of Xpert Ultra over its predecessor Xpert MTB/RIF with increased sensitivity and a slight decrease in specificity [8]. Lowering the limit of detection and introduction of the semi-quantitative ‘trace’ category with the Xpert Ultra assay has been especially beneficial in low-incidence settings or patients with paucibacillary disease [9].

Xpert Ultra has been extensively evaluated and approved as a rapid diagnostic method for TB on sputum samples [6]. Even though it has shown an improved overall performance compared to the previous version of Xpert MTB/RIF, in a subset of patients with smear-negative sputa, its sensitivity is estimated at only 63% [6]. Furthermore, TB diagnosis remains a challenge in more than a third of patients who are unable to produce sputum (sputum-scarce) [10]. In cases where TB is suspected and sputum is either non-diagnostic or scarce, there is an increased risk of delays in diagnosis and treatment. To aid in diagnosis, invasive respiratory specimens obtained by bronchoscopy are often needed. Whilst the evaluation of Xpert Ultra performance on bronchoscopy specimens has been undertaken in high-incidence areas, there are limited studies evaluating its performance in low-incidence settings [11]. The aim of our study is to evaluate the performance of Xpert Ultra for rapid detection of MTB on bronchoscopy specimens collected in a low-incidence setting in Vancouver, Canada.

## Methods

### Study design

Patients aged greater than 18 years old with suspected pulmonary TB who had undergone bronchoscopy and were tested by Xpert Ultra from March 2019 to October 2023 were included in the study. Bronchoscopy was typically performed in patients with non-diagnostic sputum (i.e. smear-negative) or those unable to produce sputum. Bronchoscopy samples were collected at two centres, an acute care tertiary care hospital and a community hospital in Vancouver, Canada. Xpert Ultra was performed on request of respirologists in patients with moderate to high risk of TB based on demographic, clinical and radiological features. We excluded patients with any sputum smear positive for AFB within 1 month prior to bronchoscopy. We did not assess patient records for extrapulmonary TB. Culture positivity for MTB was considered a reference standard for the diagnosis of confirmed TB on any sample collected by bronchoscopy.

### Specimen collection

Specimens submitted included bronchial washings and bronchoalveolar lavage (BW/BAL) and endobronchial ultrasound (EBUS) lymph node tissue biopsies. BW/BALs were collected using a flexible bronchoscope and standard collection procedures with lavage or washings of the targeted airway using sterile saline. Endobronchial lymph node tissue cultures were collected using a linear EBUS with fine needle aspirates collected from the target lymph node. If multiple samples were obtained during bronchoscopy, Xpert Ultra was most often performed on the specimen from the lung region with the greatest pathology observed on prior imaging.

### Sample processing

Bronchoscopy samples were processed using standardized laboratory procedures which included conventional digestion and decontamination using *N*-acetyl-l-cysteine-sodium hydroxide 3%. Samples were neutralized with phosphate buffer and concentrated by centrifugation followed by decanting of supernatant. This concentrate was used to make smears and inoculate media.

### AFB smear and mycobacterial culture

Auramine fluorescent smear microscopy was performed directly on concentrated samples and graded as negative or positive for AFB. AFB positive slides were graded on a standardized scale, with <5 AFB/slide graded as ‘occasional’, <4 per 10 fields with AFB and >5 AFB/slide as ‘1+’, <4 AFB/field and ≥4 per 10 fields with AFB as ‘2+’ and ≥4 AFB/field as ‘3+’. Processed specimens were inoculated onto BACTEC mycobacterial growth indicator tubes (MGITs) (Becton-Dickinson, Sparks, USA) supplemented with polymyxin b, amphotericin b, nalidixic acid, trimethoprim, and azlocillin, and Lowenstein–Jensen (LJ) medium slants (Becton-Dickinson, Sparks, USA). MGIT and LJ cultures were incubated at 37 °C for 8 weeks or until positive. Positive cultures were confirmed to be MTB using the MPT64 rapid antigen test (SC TB Ag MPT 64 Rapid, Standard Diagnostics, Inc., South Korea) and DNA probe for MTB (AccuProbe, Hologic, Inc., San Diego, USA). Positive cultures for MTB were forwarded to the provincial microbiology reference laboratory for further identification and susceptibility testing.

### Xpert Ultra

Xpert Ultra was either performed on direct samples (unconcentrated) or sediment (concentrated). In particular, Xpert Ultra was performed on unconcentrated specimens on urgent requests and prior to specimen processing. Testing was performed as per the manufacturer’s instructions. Specimens more than or equal to 0.7 ml were diluted 1 : 2 with sample reagent, whilst samples 0.5 ml to less than 0.7 ml were diluted 1 : 3 to ensure adequate testing volume. Samples were then loaded into Xpert Ultra cartridges and placed in the GeneXpert instrument. The Xpert Ultra assay produces results of either negative or positive, with positive results being further categorized as trace, very low, low, medium or high. Trace results correspond to samples that test MTB positive due to the presence of the *IS6110* and/or *IS1081* molecular signals but have an absent signal from at least three of the *rpoB* probes. Very low, low, medium or high results correspond to cycle threshold (Ct) value ranges from the first positive *rpoB* probe of 29–40, 25–28.9, 19–24.9 and 15–18.9, respectively [6].

### Statistics

Diagnostic test evaluations were done with MedCalc Software Ltd. (Version 22.016). Comparison between sample types was done via unpaired two-tailed t-test or Fisher's exact test with significance set to *P*<0.05. Average Ct values were calculated using the lowest Ct value of the four rpoB probes for each sample, as described previously [12]. The correlation between AFB smear and Ct value, and between time to positivity and Ct value, was assessed via the Spearman coefficient.

### Results

**Fig. 1.**
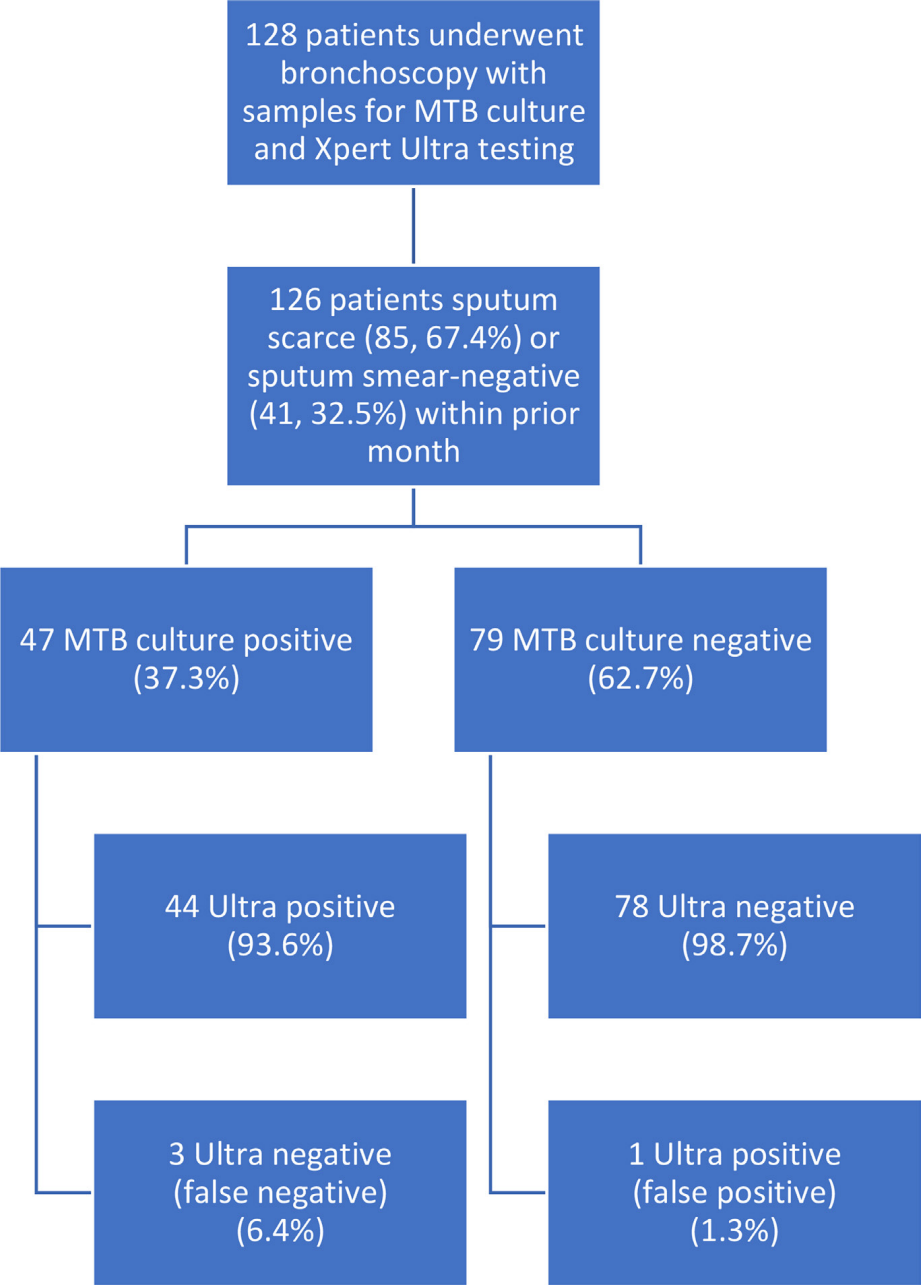
Flow diagram of study population. Inclusion criteria included adult patients (age >18), moderate to high risk for TB as per respirology assessment and sputum smear-negative or sputum-scarce (i.e. no sputum samples available for TB testing) within the past month. Twelve of the 79 MTB culture-negative patients were culture-positive for NTM. All 12 NTM culture-positive patients were Xpert Ultra negative.

During the study period, 138 bronchoscopy samples from 128 patients were analysed (Fig. 1), with ten patients having had Xpert Ultra performed on two specimens from the same bronchoscopy. Three samples from two patients with smear-positive sputa were excluded, leaving 135 samples from 126 patients. Amongst included patients, 85 (67.5%) had no prior sputum collected, whilst 41 (32.5%) sputum smears were negative. Of the 126 included patients, 47 (37.3%) were culture-positive for MTB, and another 12 (9.5%) were culture-positive for NTM (ten cases of *Mycobacterium avium complex* and two of *Mycobacterium abscessus*). Of these 59 mycobacterial culture-positive cases, 27 (45.8%) were AFB smear-positive, and 32 (54.2%) were smear-negative. Xpert Ultra and AFB smear were positive in 45 (35.7%) and 28 (22.2%) of patients, respectively. Of the MTB culture-positive patients, 44 (93.6%) were Xpert Ultra-positive, whilst 3 (6.4%) were negative. Amongst culture-negative patients, Xpert Ultra was negative in 78 (98.7%) and positive in 1 (1.3%) patient. RIF resistance was not detected in any of the positive patients or isolates, either by Xpert Ultra or phenotypic susceptibility testing. All samples were collected prior to the initiation of any anti-tuberculous therapies.

Overall sensitivity of Xpert Ultra was 93.6% (82.5%–98.7%), and specificity was 98.7% (93.2%–100.0%) with a negative predictive value (NPV) of 96.3% (89.7%–98.7%) and a positive predictive value (PPV) of 97.8% (86.2%–99.7%). In comparison, the AFB smear had a sensitivity of 40.4% (26.4%–55.7%) and a specificity of 88.6% (79.5%–94.7%). Xpert Ultra performance in all patients is described in Table 1. Of the 28 smear-positive cases, 19 (67.8%) were culture-positive for MTB, 8 (28.6%) were culture-positive for NTM and 1 was culture-negative. Amongst smear-positive patients, Xpert Ultra had a sensitivity of 100% (82.4%–100.00%) and a specificity of 100% (66.4%–100.0%). In smear-negative patients, Xpert Ultra had a sensitivity of 89.3% (71.8%–97.7%) and a specificity of 98.6% (92.3%–100%). The three false-negative Xpert Ultra results in this study came from smear-negative samples. Xpert Ultra was negative in all 12 cases of NTM.

**Table 1.** Xpert MTB/RIF Ultra performance on bronchoscopy specimens in comparison to mycobacterial culture, by patient. A total of 47 out of 126 patients were diagnosed with MTB via positive culture, whilst Xpert Ultra was positive in 45 out of 126 patients. PPA, positive percent agreement; NPA, negative percent agreement

	% (95% CI)				
	**Method**	**PPA**	**NPA**	**PPV**	**NPV**
**All patients**(*n*=126)	**Xpert Ultra**	93.6	98.7	97.8	96.3
		(82.5–98.7)	(93.2–100)	(86.2–99.7)	(89.7–98.7)
	**AFB smear**	40.4	88.6	67.9	71.4
		(26.4–55.7)	(79.5–94.7)	(51.0–81.1)	(66.1–76.2)
**AFB smear-positive**(*n*=28)	**Xpert Ultra**	100	100	100	100
		(82.4–100)	(66.4–100)	(82.4–100)	(66.4–100)
**AFB smear-negative**(*n*=98)		89.3	98.6	96.2	95.8
		(71.8–97.7)	(92.3–100)	(78.1–99.4)	(88.8–98.5)

Concentrated and unconcentrated specimens comprised 118 (87.4%) and 17 (12.6%) of specimens, respectively (Table 2). There was no difference in positivity rates, as Xpert Ultra was positive in 6 out of 17 (35.3%) of unconcentrated samples and 42 out of 118 (35.6%) of concentrated samples. The sensitivity of Xpert Ultra was lower in unconcentrated samples (75%, 95% CI=34.9–96.8) compared to concentrated (97.4%, 95% CI=86.5–99.9), although not significantly (*P*=0.07 with Fisher’s exact test). Specificity was comparable at 100% (95% CI=66.4–100) and 98.6% (95% CI=92.3–100), respectively.

**Table 2.** Xpert MTB/RIF Ultra performance on bronchoscopy specimens in comparison to mycobacterial culture, by processing technique and specimen type. BW/BALs were collected with lavage or washings of the targeted airway using sterile saline. EBUS lymph node tissue cultures were collected using a linear EBUS with fine needle aspirates collected from the target lymph node

% (95% CI)					
	**Method**	**PPA**	**NPA**	**PPV**	**NPV**
**Sediment**(*n*=118)	**Xpert Ultra**	97.4	98.6	97.4	98.6
		(86.5–99.9)	(92.3–100)	(84.4–99.6)	(90.9–99.8)
**Unprocessed**(*n*=17)		75.0	100	47.1	81.8
		(34.9–96.8)	(66.4–100)	(54.1–100)	(57.5–93.7)
**BW/BAL**(*n*=117)	**Xpert Ultra**	95.5	100	100	97.3
		(84.5–99.4)	(95.1–100)	(91.6–100)	(90.4–99.3)
**EBUS**(*n*=18)		83.3	91.7	83.3	91.7
		(35.9–99.6)	(61.5–99.8)	(42.5–97.1)	(65.3–98.5)

Sample types included 117 (86.7%) BW/BALs and 18 (13.3%) tissues from mediastinal or hilar lymph node biopsies (Table 2). Xpert Ultra was positive in 42 (35.9%) BW/BALs and 6 (33.3%) EBUS samples. Overall, Xpert Ultra had a sensitivity of 83.3% (95% CI=35.9%–99.6%) and a specificity of 91.7% (95% CI=61.5%–99.8%) on lymph node samples, compared to a sensitivity of 95.5% (95% CI=84.5%–99.4%) and a specificity of 100% (95% CI=95.1%–100%) on BW/BAL samples. Three patients with positive MTB culture had Xpert Ultra performed on both BW/BAL and EBUS samples. In two of these patients, Xpert Ultra had lower semi-quantitative categories from the EBUS sample compared to BW/BAL (‘low vs medium’, ‘trace vs medium’). In the other patient, the results of Xpert Ultra were in the ‘high’ category for both BW/BAL and EBUS samples. On the other hand, the single false-positive result by Xpert Ultra came from a ‘trace’ positive EBUS with a corresponding negative BW/BAL. Of the positive, non-trace Xpert Ultra results in this study, there was no significant difference between average first positive Ct values from BW/BAL samples and lymph node biopsies (23.0+/−4.7 vs 21.8+/−3.7, *P*=0.64).

**Fig. 2.**
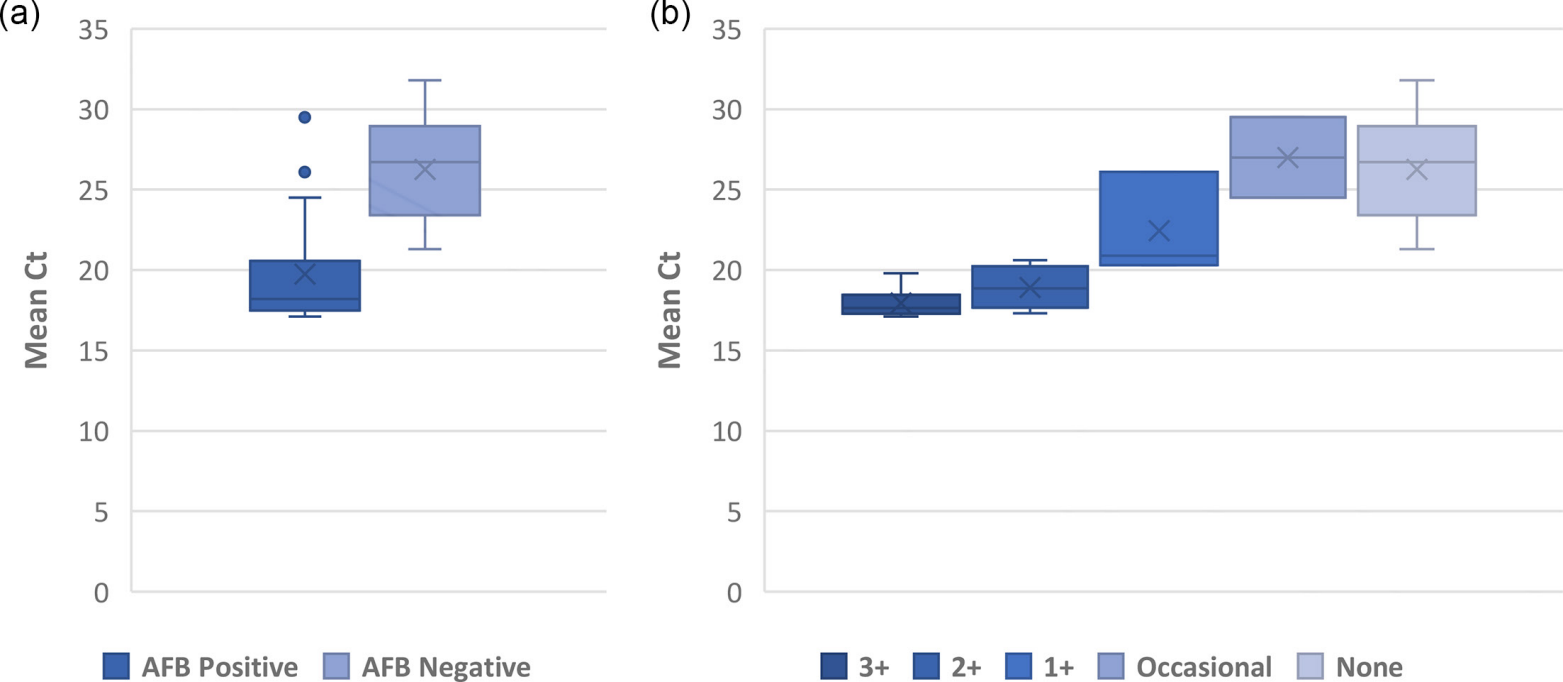
Xpert Ultra Ct values compared to AFB smear. (a) Box and whisker plot of Xpert Ultra Ct values from AFB-smear positive (n=21) vs AFB-smear negative (*n*=20) samples (19.7+/−3.2 vs 26.3+/−3.2, *P*<0.001 by t-test). ‘Trace’ Xpert Ultra results excluded. Boxes span the interquartile range (IQR) of the data, with a line representing the median. Whiskers represent data within up to 1.5 times the IQR. Outliers are represented by individual points. (b) Box and whisker plot of Ct values by semi-quantitative smear microscopy grade (Spearman coefficient of 0.83, *P*< 0.001). AFB were graded on a standardized scale, with <5 AFB/slide graded as ‘occasional’, <4 out of 10 fields with AFB and >5 AFB/slide as ‘1+‘, <4 AFB/field and ≥4 out of 10 fields with AFB as ‘2+‘ and ≥4 AFB/field as ‘3+‘.

Semi-quantitative smear results correlated strongly with semi-quantitative Xpert Ultra results. Of the 135 specimens included in this study, 48 were Xpert Ultra-positive, including 21 (43.8%) from smear-positive samples and 27 (56.3%) from smear-negative samples. Amongst the 27 smear-negative samples, Xpert Ultra-positive samples, there were 7 ‘trace’, 8 ‘very low’ and 12 ‘low’ results. Whilst amongst the 21 smear-positive samples, Xpert Ultra-positive samples, there were 1 ‘very low’, 4 ‘low’, 5 ‘medium’ and 11 ‘high’ results. Excluding the ‘trace’ results, as they do not produce an *rpoB* Ct value, the average Xpert Ultra Ct value from the smear-positive samples was 19.7+/−3.2, significantly lower than the average Ct value of the smear-negative specimens at 26.3+/−3.2 (Fig. 2a, *P*<0.001). There was a strong association between semi-quantitative smear grades and corresponding Ct values, with a Spearman coefficient of r=0.83 (Fig. 2b, *P*<0.001).

**Fig. 3.**
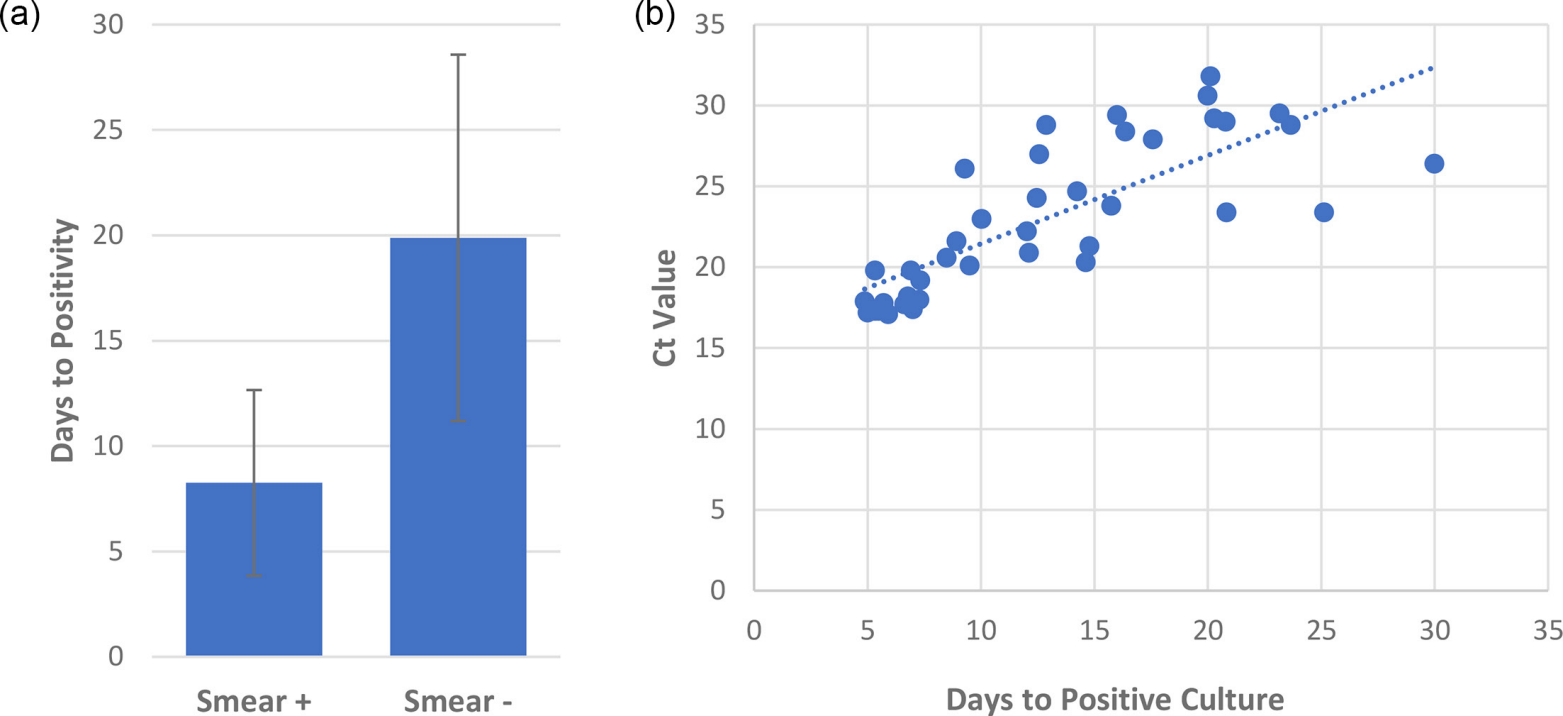
Correlation between Xpert Ultra and time to positivity of MTB culture. (a) Days to positive culture of AFB smear-positive (*n*=19) and smear-negative patients (*n*=28) (8.3+/−4.4 vs 19.9+/−8.7, *P*<0.001 by unpaired, two-tailed t-test). Error=sd. (b) Correlation between Xpert Ultra Ct values and days to positive culture (Spearman correlation of 0.85, *P*<0.001). +, positive; −, negative.

The average time to positive MTB culture was 15.2+/−9.2 days, whilst AFB smear and Ultra results were available within 24 h. The mean time to positivity of culture amongst smear-positive and Xpert Ultra-positive was 8.3+/−4.4 days versus smear-negative and Xpert Ultra-positive of 19.9+/−8.7 days (Fig. 3, *P*<0.001). Meanwhile, time to positivity for Xpert Ultra false-negative samples was 30.2+/−10.3 days. Furthermore, Xpert Ultra Ct values and time to positivity (days) had a strong correlation with a Spearman coefficient of 0.85 (Fig. 3, *P*<0.001).

## Discussion

Diagnosis of TB in sputum-smear-negative and sputum-scarce patients presents a challenge. Our study demonstrates enhanced diagnosis of TB in more than a third of patients who had undergone bronchoscopy. Xpert Ultra on bronchoscopy and EBUS specimens had excellent performance in moderate- to high-risk patients with an overall sensitivity of 93.6% and a specificity of 98.7%. Although there was a 100% sensitivity amongst smear-positive samples, sensitivity in smear-negative samples was 89.3%. A recent systematic review reported a sensitivity of 90.9% and a specificity of 95.6% of Xpert Ultra in the detection of pulmonary TB compared to culture, with a sensitivity of 77.5% and a specificity of 95.8% in smear-negative participants [7]. However, this review included all respiratory samples and did not separate sputum from bronchoscopy samples. A study by Mekkaoui *et al*. similarly found an overall sensitivity and specificity of 88.2% and 94.7%, respectively, in bronchoscopy samples from a low-incidence setting in Brussels, Belgium [13]. In studies from high-incidence settings, sensitivities ranged from 88.9% to 97.4% [14,15].

In addition to the incidence of TB in various studies, the selection criteria play a crucial role in test performance characteristics. For instance, in our study, cases were reviewed by respirologists, and patients in whom sputum was non-diagnostic or unobtainable and whose risk for TB was moderate to high were included in the study. This selection process is reflected in the high positivity rate of TB in our study when compared to other studies that include all patients regardless of their TB risk. For example, Mekkaoui *et al*. found a culture positivity rate of 3% and Xpert Ultra PPV of 34.1%, whereas in our study, the culture positivity rate was 37.3% with a PPV of 97.8% [13]. Other studies conducted in low-incidence settings similarly demonstrated an enhanced culture positivity rate and improved performance when patient selection was driven by clinical suspicion for TB [9,16].

In addition to patient selection, a range of reported sensitivities can be attributed to the choice of comparator (clinical diagnosis of TB vs culture-confirmed TB), type of specimen (bronchial aspirates/washings versus BAL), sampling method and specimen handling (decontamination, concentration, etc.). For instance, in most studies when a comparison is made against a clinical reference standard, sensitivities of Xpert Ultra tend to be lower than when compared to culture. In a systematic review of the performance of Xpert MTB/RIF (original) on bronchoscopy samples, performance was much improved if the chosen comparator was culture versus composite reference standard which included clinical diagnosis, with sensitivities of 87% and 69%, respectively [17]. Furthermore, the recovery of MTB is higher from peripherally collected, higher-volume BAL samples than more proximal, lower-volume BW in sputum-negative or sputum-scarce cases, making BAL the preferred specimen [18]. A limitation of our study is that samples were a combination of these two techniques, and we were unable to directly compare them.

In addition to being able to obtain deeper specimens and achieve earlier diagnosis in patients with smear-negative sputum or sputum-scarce pulmonary TB, bronchoscopy further allows visualization of airways and performance of lymph node biopsies using EBUS. In our study, Xpert Ultra on EBUS samples had a lower sensitivity compared to BW/BAL specimens. The reduced performance could be related to specimen collection issues, as sampling of lymph nodes via fine needle biopsy retrieves only a small amount of tissue. However, our interpretation is limited due to the small number of lymph node biopsies in our study. Yao *et al*. found a sensitivity of 90.9% and specificity of 59.6% of Xpert Ultra on EBUS biopsy specimens compared to culture [16]. The only false-positive Xpert Ultra result in our study was from a biopsy specimen in a patient who was not previously treated for TB, which could potentially represent subclinical or latent TB [19].

Although we observed a trend towards lower sensitivity in unconcentrated over concentrated BW/BAL specimens, our sample size of unconcentrated samples was too small to reliably explore this difference. Although the concentration step should enhance diagnostic yield, specimen processing can also lead to loss of mycobacterial viability. Previous studies have shown mixed results on whether there is a detectable difference between processed and unprocessed results on the original Xpert MTB/RIF assay [20]. According to the manufacturer, Xpert Ultra can be used with either concentrated or unconcentrated sputum with different dilution protocols. Further work is needed to investigate how the processing step affects Xpert Ultra’s performance on bronchoscopy specimens.

As expected, Ct values from Xpert Ultra had a strong correlation to a bacillary burden as measured by AFB smear grading. This has been demonstrated previously in sputum samples, leading to considerations of using the Xpert platform as a screening tool to guide isolation measures [21]. Smear AFB grade in sputum is known to correlate with infectious potential [22], but it is unclear whether the same is true with smears from bronchoscopy samples. At the very least, Xpert Ultra categorization provides an estimate of disease burden that correlates with microscopy. Similarly, Xpert Ultra categorization, based on Ct value ranges, correlated with time to culture positivity, with higher categories leading to earlier growth in culture. Others have similarly reported that the Xpert Ultra category had a strong correlation with the time of MGIT growth [23]. In our study, all the samples with Ct values of <20 became positive within 7 days, reflective of high bacillary burden.

Limitations of our study include the limited applicability to settings lacking access to bronchoscopy procedures. Bronchoscopy is an invasive procedure requiring specialized infrastructure and expertise, therefore limited to hospitals and higher-resource settings. However, in low-incidence settings where the diagnosis of TB is infrequent, it represents an important adjunct to diagnosis. Additionally, specimens obtained via BAL and BW were grouped together in our study, rather than compared to each other as discussed previously. We also did not review cofounders that are known to impact results from the Xpert Ultra test, such as HIV status or previous TB infection. However, Zhang *et al*. found a sensitivity of 96% and specificity of 92% for bronchoscopy samples from patients living with HIV in China, suggesting good performance from bronchoscopy samples in that population [24]. We did not detect any RIF resistance in our study, as rates of drug-resistant MTB in our area are low. Notably, each isolate was confirmed to be RIF susceptible in subsequent testing at a reference laboratory, confirming that Xpert Ultra did not miss any resistant cases.

In summary, Xpert Ultra performance on bronchoscopy specimens demonstrated high sensitivity and specificity in comparison to culture in a Canadian setting. The test remains reliable even in smear-negative samples with a slight drop in performance. Our results suggest that Xpert Ultra is a useful adjunct in high-risk patients with non-diagnostic sputum or in whom sputum is unobtainable. Pre-test probability and careful patient selection play an important role in enhancing the diagnostic performance of Xpert Ultra in a low-incidence setting.

## Abbreviations

AFB: acid-fast bacilli
BAL: bronchoalveolar lavage
BW: bronchial washings
Ct: cycle threshold
EBUS: endobronchial ultrasound
LJ: Lowenstein–Jensen
MGITs: mycobacterial growth indicator tubes
MTB: *Mycobacterium tuberculosis*
NPA: negative percent agreement
NPV: negative predictive value
NTM: non-tuberculous mycobacteria
PPA: positive percent agreement
PPV: positive predictive value
RIF: rifampin
TB: tuberculosis
